# Exosomes as Novel Diagnostic Biomarkers and Therapeutic Tools in Gliomas

**DOI:** 10.3390/ijms241210162

**Published:** 2023-06-15

**Authors:** Panagiotis Skouras, Antonios N. Gargalionis, Christina Piperi

**Affiliations:** 1 Department of Neurosurgery, ‘Evangelismos’ Hospital, Medical School, National and Kapodistrian University of Athens, 10676 Athens, Greece; panskouras@med.uoa.gr; 2 Department of Biological Chemistry, Medical School, National and Kapodistrian University of Athens, 11527 Athens, Greece; agargal@med.uoa.gr; 3 Department of Biopathology, ‘Eginition’ Hospital, Medical School, National and Kapodistrian University of Athens, 11528 Athens, Greece

**Keywords:** exosome, brain tumors, glioma, glioblastoma, extracellular vesicles, drug delivery

## Abstract

Exosomes constitute small extracellular vesicles that contain lipids, proteins, nucleic acids, and glycoconjugates from the secreted cells and are capable of transmitting signals between cells and coordinating cellular communication. By this means, they are ultimately involved in physiology and disease, including development, homeostasis, and immune system regulation, as well as contributing to tumor progression and neurodegenerative diseases pathology. Recent studies have shown that gliomas secrete a panel of exosomes which have been associated with cell invasion and migration, tumor immune tolerance, potential for malignant transformation, neovascularization, and resistance to treatment. Exosomes have therefore emerged as intercellular communicators, which mediate the tumor–microenvironment interactions and exosome-regulated glioma cell stemness and angiogenesis. They may induce tumor proliferation and malignancy in normal cells by carrying pro-migratory modulators from cancer cells as well as many different molecular cancer modifiers, such as oncogenic transcripts, miRNAs, mutant oncoproteins, etc., which promote the communication of cancer cells with the surrounding stromal cells and provide valuable information on the molecular profile of the existing tumor. Moreover, engineered exosomes can provide an alternative system for drug delivery and enable efficient treatment. In the present review, we discuss the latest findings regarding the role of exosomes in glioma pathogenesis, their utility in non-invasive diagnosis, and potential applications to treatment.

## 1. Introduction

Extracellular vesicles play a notable role in physiological conditions and disease [1]. They are divided into different vesicle types according to their origin, size, protein markers and content. The main extracellular vesicle types are apoptotic bodies, microvesicles, and exosomes. Apoptotic bodies and microvesicles can be large vesicles, usually over 500 nm in size, resembling the respective apoptotic and viable cells in composition. On the contrary, exosomes represent smaller vesicles with a shape size that varies between 30 and 200 nm [2]. Exosomes are small, single-membraned secreted organelles. They exhibit common topology with the cell and mainly include secreted proteins, glycoconjugates, lipids, and nucleic acids [3,4,5]. They can be generated from the endolysosomal pathway through intraluminal budding and the fusion of the multivesicular bodies with the plasma membrane [2].

Exosomes are also considered as cellular waste products, serving as signaling vesicles between cells and coordinating cell communication via the transport of proteins and nucleotides [6]. The uptake of exosomes depends on the accepting cell type and takes place mainly via endocytosis or phagocytosis [7,8].

Furthermore, exosomes participate both in health and disease [1]. They are involved in pathological and physiological mechanisms, such as homeostasis, development, and immune response [9]. They function to remodel the extracellular matrix, transmit signals and molecules to other cells. By this means, they affect immune surveillance and the inflammation process, the coagulation cascade, and stem cells’ maintenance and plasticity, as well as repair of tissue damage [10,11,12]. A representative example of their function is the Juno protein, which is eliminated via exosomes after fertilization to avoid polyspermy [13]. However, exosomes also participate in pathological conditions. They support tumor growth, induce an immune response against autoantigens in the context of autoimmune diseases, transfer harmful proteins in prions’ diseases, cause viral load during HIV-1 infection, and are also linked with the onset of neurodegenerative diseases [14,15,16,17,18].

Brain tumors present molecular heterogeneity and have been related to high mortality. Gliomas present the most frequent primary CNS tumors, characterized by aggression, recurrence, and mortality [19]. They specifically represent 24.7% of all primary brain tumors and 74.6% of malignant brain tumors [20]. Grade 4 astrocytoma, known as GBM, is the most invasive, aggressive adult brain tumor, with dismal prognosis even after aggressive therapeutic schemes employing surgical resection followed by radiotherapy and Temozolomide (TMZ) administration. GBM is characterized by increased invasive capacity and high probability of relapse after surgery. Drug penetration is limited at the tumor site and resistance to chemotherapy develops rapidly, leading to reduced survival [21,22].

**Figure 1:**
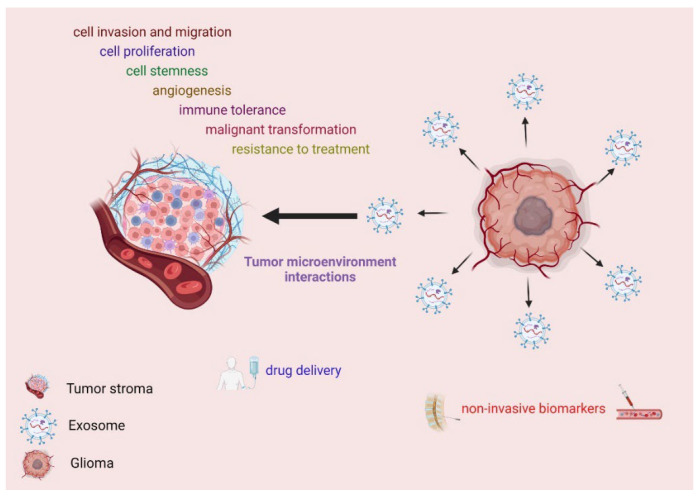
The role of exosomes in glioma pathogenesis and treatment. Exosomes derived from glioma cells mediate the interactions of cancer cells with the tumor microenvironment and facilitate certain malignant properties. Exosomes can be used as diagnostic and therapeutic tools since they can be non-invasively detected in blood samples and cerebrospinal fluid. They have been also suggested as potential drug delivery systems (figure created using BioRender.com, accessed on 16 May 2023).

The interaction of primary and metastatic brain tumors with their microenvironment is critical for cancer development and sensitivity to therapy. Cancer cells secrete a variety of exosomes in the extracellular space, the cargo of which consists of functional biomolecules (Figure 1) [23]. Initially, exosomes were investigated as therapeutic non-cellular antigens to produce vaccines against tumors or infectious diseases [24,25]. In this context, extracellular vesicles (EVs) emerge as intercellular communicators, which mediate the tumor–microenvironment interactions (Figure 1) [26].

Exosomes are of particular importance in brain tumors, especially in GBM, since recent studies demonstrate that this specific type of tumors secrete a variety of exosomes which have been associated with cell invasion and migration, tumor immune tolerance, potential for malignant transformation, neovascularization, and resistance to treatment (Figure 1) [27].

In the present review, we discuss biogenesis and the general functions of exosomes, methods of exosomes isolation, specific exosome-mediated modifications in gliomas, and exosome-mediated interactions of brain tumor cells with stroma, including their tumor-promoting immunomodulatory role. We also discuss exosome-regulated glioma cell stemness and angiogenesis as well as experimentally established exosome biomarkers in gliomas. Furthermore, we highlight the involvement of exosome secretion and function in respect to mechanisms of therapeutic efficacy or resistance in these types of brain tumors.

## 2. Biogenesis and Functions of Exosomes

Extracellular vesicles contain a lipid bilayer membrane which encloses variable genetic material, cell debris, and proteins. Every EV has its size, characterized by a unique biogenesis process, function, and related mechanisms. EVs are distinguished into three subgroups: ectosomes or shedding microvesicles (SMVs), exosomes, and apoptotic bodies [8,28]. They play a crucial role in organogenesis and tissue homeostasis; their cargo reflects the origin of the tissue while higher numbers of EVs are released by cancer cells [29].

**Figure 2:**
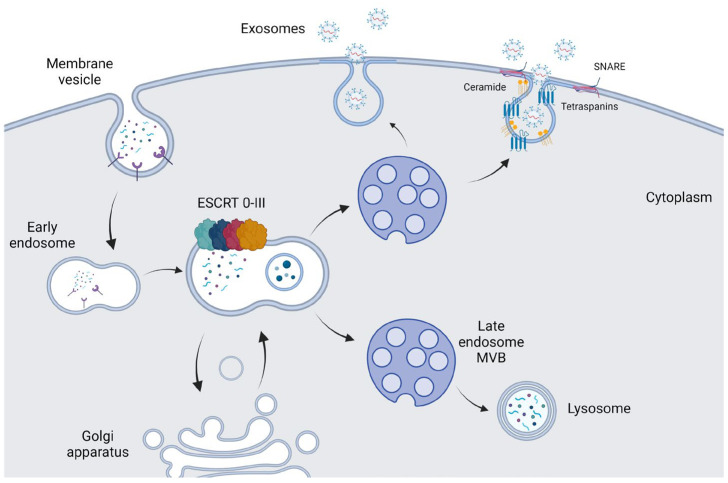
The exosome biogenesis pathway. Early endosomes could follow the ESCRT-dependent exosome pathway to form MVBs. MVBs can either follow the lysosomal pathway or form and secrete exosomes. There are also alternative ESCRT-independent ways of exosome biogenesis though ceramide, tetraspanins, and SNARE proteins. ESCRT, endosomal sorting complex required for transport; MVB, multivesicular bodies; SNARE, SNAP receptor (figure created using BioRender.com, accessed on 7 June 2023).

Exosomes are vesicles of endosomal origin. They are produced from vesicles budding into endosomes. The early endosome is formed through the inwards invasion of the plasma membrane, while the late endosome membrane grows further to form small vesicles, leading to the formation of multivesicular bodies (MVB). The intraluminal vesicles (ILVs) are shaped inside the MVBs via the invagination of the inner endosomal membrane. ILVs incorporate cytoplasmic inclusions and transmembrane and peripheral proteins. ILVs will either fuse with the lysosome that will cause the degradation of the vesicles or will fuse with the plasma membrane that will lead them to the extracellular space through exocytosis. The vesicles that have been secreted through the later process are known as exosomes (Figure 2) [30].

More specifically, the production of ILVs is facilitated by the endosomal sorting complex required for transport (ESCRT) process, which involves four separate protein ESCRTs (0 through III). ESCRTs function in coordination to facilitate loading cargo to ILVs [30]. The components of the ESCRT machinery recognize ubiquitinated proteins via the specific binding units of the ESCRT-0 complex. Following the interactions of the ESCRT complexes, ESCRT-III ultimately mediates the budding process (Figure 2) [31]. However, there are also ESCRT-independent mechanisms to enable the formation of MVB, vesicle budding, and protein cargo sorting. Alternative processes of exosome production involve the lipid molecule ceramide [32], small GTPases [33], and the SNAP receptor (SNARE) family of proteins (Figure 2) [34].

Exosomes can also occur through direct budding from the plasma membrane [13,35]. They can be released after budding into intracellular plasma membrane-connected compartments (IPMCs). IPMCs represent the deep invaginations of the plasma membrane, which cannot be easily distinguished from MVBs. IPMCs form long necks in conjunction with the extracellular environment and need to become deconstructed to release the vesicles [5]. Exosome size, shape and density are highly variable and mostly depend on the types of proteins, lipids, enzymes, and minerals [3,36,37].

Exosomes are heterogenous in composition. They are enriched by a variety of proteins found on the surface, in membrane-associated protein complexes and soluble proteins in the exosome lumen. They are rich in tetraspanin proteins (cluster of differentiation 81 (CD81), CD82, CD37, CD63, CD9) [38,39], virus-encoded membranes and envelope proteins [40,41,42], integral signaling proteins (vascular endothelial growth factor receptor 2 (VEGFR-2), epidermal growth factor receptor (EGFR), cytokine receptors and G-protein coupled receptors (GPCRs), c-Src, insulin-like growth factor 1 (IGF-1) receptor, focal adhesion kinase (FAK)) [43,44,45], lipid-anchored outer membrane proteins, extracellular matrix proteins, peripheral surface proteins, lipid-anchored inner membrane proteins (small guanosine triphosphate (GTP)ases), scaffold proteins, and enzymes [5]. Exosomes also contain glycoconjugates and lipids, RNAs (mRNAs and microRNAs), and DNA [46,47,48].

Exosomes exhibit notable roles in various physiological processes, including immune surveillance and inflammation, coagulation cascade, stem cells’ maintenance and plasticity, and the repair of tissue damage [10,11,12]. They mainly demonstrate signaling properties by trafficking bioactive molecules, such as microRNAs, mRNAs, proteins, and lipids. Exosomes are secreted by B lymphocytes, macrophages, and dendritic cells. Therefore, they participate in antigen presentation and the transfer of antigens and major histocompatibility complex (MHC) molecules, thereby participating in the regulation of the immune response. Exosomes can also activate the receptors of the cell membrane by transferring protein and lipid ligands. They can also transfer cell surface receptors, transcription factors, oncogenes, mRNAs, and miRNAs to target cells [49]. In addition, exosomes are secreted by microglial cells into the CNS to regulate brain inflammation after CNS injury but are also secreted by oligodendrocytes, Schwann cells, astrocytes, and neurons [49].

## 3. Isolation Methods of Exosomes

Ultracentrifugation (UC) is the most common technique for exosomes isolation in most cancer types, yielding significant amounts [50,51]. However, it is rather demanding due to their low density and small sizes [52,53]. For example, in non-small-cell lung cancer (SK-MES-1) cell cultures, 1.3 × 10^12^ particles/mL were isolated with UC from 150 mL cell culture medium (CCM), and in human primary GBM (U-87 MG) cells, 10^12^ particles/mL were isolated from 280 mL CCM [52,54]. Another method of isolation, depending on size, is ultrafiltration (UF), which is faster and cheaper than UC, but it presents low efficacy (since vesicles can be trapped within the sieve pores) and low specificity (since similar particles to exosomes can also be filtered) [55].

Immunoaffinity isolation technique employs the specific interaction of membrane-bound EV surface markers that are expressed on the exosome surface, serving as receptors with specially designed antibodies acting as ligands [51]. In this way, exosomes can be highly purified and subtyped, but this method can be expensive, and the manufacturing of specific antibodies is still under development [56]. Water-excluding polymers, such as polyethylene glycol (PEG), can also be used to induce exosomes release by modifying their solubility and dispersibility [51]. By leveraging the biochemical and physical properties of exosomes, such as immunoaffinity, density, size, and microfluidics technology, enables the rapid and efficient isolation of microscale EVs while reducing the required sample volume and reagent consumption [51] (Table 1).

## 4. Exosome-Mediated Modifications in Gliomas

Exosomes induce tumor proliferation and malignancy in normal cells by carrying pro-migratory modulators from cancer cells [57]. Nanoscale peak force imaging was employed in GBM-derived exosomes showing the presence of surface nanofilaments for the first time. These nanofilaments were detected on the surface of glioblastoma exosomes, presenting unique biophysical properties. They were stiffer and more adhesive, exhibiting increased cellular uptake compared to normal cell-derived exosomes, thus facilitating cellular binding, the uptake of exosomes, and intercellular communication in GBM [36].

Studies have shown that astrocytes that have been modified by exosomes (deriving from GBM cells) become tumorigenic [58,59]. It has been reported that a single GBM cell can produce almost 10.000 EVs in approximately 48 h, which enables them to exhibit tumor cell invasion and migratory phenotype. Furthermore, exosomes increase immune tolerance, enhance chemotherapy resistance, and induce vascular supply of GBM [27,60].

GBM exosomes are characterized by a wide variety of molecular cancer modifiers, such as oncogenic transcripts, miRNAs, mutant oncoproteins, etc., which promote the interplay among cancer cells and the surrounding normal cells [61,62].

Exosomes carry miRNAs and other compounds which can reflect the progression of different brain pathologies, suggesting that exosomes may reflect the molecular profile of the existing tumor [63]. Exosomes as suitable carriers for miRNAs have been shown to transport several miRNAs (miR-21, miR-29a, miR-221, and miR-222, etc.) in several in vitro studies and microarrays analyses, which can trigger proliferation and inhibit the apoptosis of GBM cells [64]. In this way, exosomes may present elegant delivery vectors for tumor-suppressive miRNAs against gliomas [65]. Approximately 1000 proteins were identified with a mass spectrometry analysis in GBM exosomes, which are pro-angiogenic factors (interleukin-6 (IL-6), IL-8, angiogenin, tissue inhibitor of metalloproteinase-1 (TIMP-1) and TIMP-2) in endothelial cells of normal brain and enhance malignancy by causing hypoxia [66,67].

Exosomes can reach unaffected stromal cells and receptors that have tumorigenic features, such as EGFRvIII, human epidermal receptor (HER2), and platelet-derived growth factor receptor (PDGFR), favoring GBM proliferation [64]. Furthermore, exosomes carry the phosphatase and tensin homolog (PTEN), which can be found in the cytoplasm or in the nucleus, and its absence has been correlated with tumorigenesis [68]. Nedd4 Family Interacting Protein 1 (Ndfifip1) participates in the internalization of exosomes, the cargo of which is PTEN. Interestingly, Ndfifip1 is suppressed in GBM, with PTEN intranuclear concentration being subsequently repressed and allowing tumor cells to proliferate and survive [64].

Another imperative characteristic of GBM is their capacity to use invadopodia, by which they take over the adjacent cells (astrocytes). Invadopodia are membrane-derived extensions of GBM cells, which adhere to the neighbor tissue and facilitate proteolytical degradation [69,70]. Numerous proteins released by exosomes derived from GBM are linked to the formation of invadopodia and the consequent ability of GBM to invade, including integrin β1 (ITGB1), Annexin A1 (ANXA1), actin-related protein 3 (ACTR3), calreticulin (CALR), and programmed cell death 6-interacting protein (PDCD6IP), as well as others [70]. Hallal et al. observed podosome formation in astrocytes and matrix destruction, after the interaction with exosomes deriving from GBM cells. This process is enhanced by decreased levels of p53 and consequently exosomes promote tumorigenesis and induce the neighboring astrocytes to become tumorigenic [58].

## 5. Exosome-Mediated Interactions of Brain Tumor Cells with Stroma

The microenvironment of the brain includes astrocytes, microglia, neurons, endothelial cells, immune cells, extracellular matrix (ECM) of distinct composition, and the blood–brain barrier. In cancer, exosomes mediate intercellular communication as well as the interplay of cancer cells and their microenvironment in order to promote tumor progression [26].

### 5.1. Tumor-Promoting Immunomodulatory Role of Exosomes

GBM cells are surrounded by nonmalignant stromal cells, including astrocytes, ependymal cells, oligodendrocytes, and microglia. They are also surrounded by infiltrating immune cells, such as myeloid-derived monocytes/macrophages and lymphocytes [71]. GBMs can generate immunosuppressive functions in the tumor microenvironment. This intercellular communication is mediated via tumor-derived exosomes, which carry various immune-modulating molecules [72]. The clinical efficacy of anticancer agents is limited by the tumors’ immunosuppressive mechanisms.

These mechanisms are mediated by regulatory T cells, tumor-associated macrophages, and myeloid-derived suppressor cells (MDSCs). It is also known that brain tumor-initiating cells (BTICs) are stem-like cells, which offer GBM resistance to chemotherapy and radiotherapy. BTICs exert their action by exporting extracellular matrix protein tenascin-C (TNC) via exosomes to T cells, thereby suppressing T cell activity [73]. Regarding the role of MDSCs, it has been observed that hypoxia upregulates the release of glioma-derived exosomes, which facilitate the immunosuppressive function of MDSCs through miR-29a and miR-92a. First, miR-29a exerts its action on MDSCs by targeting high-mobility group box transcription factor 1 (Hbp1), while miR-92a modulates the activity of protein kinase cAMP-dependent type I regulatory subunit alpha (Prkar1a) [74]. The secretion of glioma exosomes and cerebrospinal fluid (CSF)-derived glioma exosomes are rich in miR-1246. More specifically, hypoxia upregulates miR-1246 via the upregulation of heterogeneous nuclear ribonucleoprotein A1 (hnRNPA1) and POU class 5 homeobox 1 (POU5F1). miR-1246 upregulation induces the activation and differentiation of MDSCs through phosphatase 3 (DUSP3)/extracellular signal-regulated kinase (ERK) [75]. Exosomes which are rich in antigen-presenting molecules and tumor antigens can induce dendritic cells activation. The induction of dendritic cells transforms T cells to become glioma specific CD8^+^ cytotoxic T lymphocytes [76]. The glioma-specific isocitrate dehydrogenase (IDH) mutation is detected in tumor-derived glioma small extracellular vesicles. The vesicles bearing the IDH mutation have been detected to be more immunosuppressive than wild-type EVs [77].

Exosomes secrete cytokines which support tumor growth. Tumor necrosis factor-alpha (TNF-α) and Interleukin-1 beta (IL-1β) can induce the production of the small heat shock protein, CRYAB (HspB5) in U373 glioma cells, and U373-secreted exosomes. CRYAB has anti-apoptotic activity and therefore facilitates the survival of tumor cells. Treatment with TNF-α and IL-1β also transforms the proteome of the secreted exosomes, which is enriched with cancer-promoting protein molecules [78]. The treatment of healthy donors’ peripheral blood mononuclear cells (PBMCs) with exosomes generated from glioma stem cells leads to the suppression of T cell activation, proliferation, and production of Th1 cytokines. The cytokine profile induced by glioma stem cells (GSC)-derived exosomes resembles that of monocytic MDSCs [79].

### 5.2. Exosome-Regulated Glioma Cell Stemness and Angiogenesis

The implication of the exosome-regulated stemness of glioma cells and exosome-regulated hypoxia-induced angiogenesis have been already highlighted as features of exosome-mediated tumor-promoting immunomodulation. It has been documented that EVs from human glioma-derived stem-like cultures (GSCs), among them exosomes, contain extracellular RNA complexes, which reliably reflect the intracellular transcriptome [80]. When GSCs are treated with exosomes enriched in miR-124a, this leads to the decreased viability and clonogenic capacity of GSCs. In accordance, in vivo treatment leads to the decreased survival of preclinical models. This reduction is mediated through the miR-124a-targeted suppression of Forkhead box (FOX)A2 [81]. Experiments in the co-cultures of bulk tumor cells and brain tumor spheroid-forming cells (BTSCs) from medulloblastomas demonstrate a distinct miRNA profile with high expression in both BTSC cells and respective exosomes. miR-135b and miR135a were the most abundant of the miRNAs, both targeting angiomotin-like 2 (AMOTL2). The low expression of AMOTL2 has been correlated to low survival in pediatric Group 3 and Group 4 medulloblastoma patients [82]. Experiments in GSCs derived from U-251 cells show that when the GSCs become transfected with miR-21 mimics, they produce exosomes. In co-cultures with human brain endothelial cells mir-21-transfected exosomes upregulate VEGF, as well as miR-21 expression in GSCs and endothelial cells. This can also promote the angiogenic capacity of endothelial cells; thus, GSCs facilitate endothelial cells’ ability to promote angiogenesis through miR21/VEGF signaling [83].

EVs with exosome characteristics isolated from GBM patients’ plasma have been found abundant in proteins that regulate hypoxia, such as pentraxin 3 (PTX3), matrix metalloproteinase 9 (MMP9), platelet-derived growth factor (PDGF)AB/AA, interleukin-8 (IL-8), CD26, plasminogen activator inhibitor 1 (PAI1), and caveolin 1 (CAV1). Hypoxia-related mRNAs are significantly overexpressed in the hypoxic sites of GBM mouse xenografts. IL-8, a hypoxia-induced cytokine involved in the formation of aggressive gliomas, demonstrates increased expression levels (approximately 3.4-fold compared with control mice) in exosomes from GBM tumor-bearing mice and has been also linked with hypoxic regions of GBM xenografts [84,85]. Furthermore, it has been shown that hypoxia favors autophagy and polarization of tumor-associated macrophages (TAMs). The underlying mechanism involves the IL-6-p (Interleukin 6 phospho) signal transducer and activator of transcription (STAT) 3-miR-155-3p-autophagy-pSTAT3 positive feedback loop. The facilitation of autophagy and TAM polarization through this loop leads to the promotion of proliferation and migration in vitro and in vivo [86]. Furthermore, hypoxia-stimulated glioma-derived exosomes regulate the capacity of MDSCs. Mechanistically, MDSCs expansion is induced by the expression of miR-10a and miR-21 in hypoxic exosomes by regulating RAR-related orphan receptor alpha (RORA) and PTEN [87]. lincPOU3F3 is upregulated in human glioma tissues, and several experiments have been conducted in exosomes derived from linc-POU3F3 shRNA-treated A172 cells (shA172-Exo) and A172 cells (A172-Exo). When human brain microvascular endothelial cells (HBMECs) were cultured with exosomes, they exhibited higher lincPOU3F3 expression and A172-exosomes were shown to promote HBMECs proliferation, migration, tubular-like structure development in vitro, and arteriole generation in vivo compared to shA172-Exo with silenced lincPOU3F3 expression [88].

### 5.3. Exosome Biomarkers in Gliomas

Exosomes contain a variety of molecules that transfer information between brain tumor cells; therefore, they have been highlighted as promising biomarkers. It has been proposed that aggressive GBM cells increase intracellular calcium, and, in turn, this leads to a further increase in exosome secretion. There is a distinct expression profile in the EVs of GBM cell lines that correlates with cell invasion [70]. Exosomes can cross the intact blood–brain barrier (BBB) and, along with other extracellular vesicles, can be detected in the peripheral blood of an orthotopic xenotransplant mouse model of human glioma-cancer stem cells. Furthermore, in the periphery of human glioma patients, the IDH1G395A biomarker can be detected in EVs, thereby offering a non-invasive way of monitoring glioma progression [89]. In another orthotopic xenograft mouse GBM model, both brain and blood exosomes were analyzed. Both types of exosomes share high Dynamin-3 (DNM3) and p65 expression, along with decreased p53 expression. This observation suggests that DNM3, p65 and p53 could serve as potential biomarkers derived from exosomes in GBM [90]. High-grade gliomas exhibit increased the expression of the oncogenic epidermal growth factor receptor variant III (EGFRvIII). The analysis of serum showed that EGFRvIII can be non-invasively detected in exosomes with an overall clinical sensitivity of 81.58% and specificity of 79.31%, respectively [91]. EGFRvIII expression alters the properties of EVs enriched in exosomes and the expression of EVs-related genes [92]. Furthermore, EVs from CSF can also reflect the status of GBM EGFRvIII variant and wild-type EGFR [93]. Oncogenic EGFRvIII can be transferred between glioma cells via membrane microvesicles leading to the activation of oncogenic pathways [43]. Human and mouse GBM extracellular nanovesicles that resemble exosomes also contain the active *Ras* oncogene and a variety of signaling cascade components [94].

Non-coding RNAs and miRNAs, especially, have also been detected in the exosomes of GBM origin. Primary GBM cells derived from the respective tumors secrete exosomes up to 15 passages. These exosomes contain a variety of mRNAs and miRNAs and proteins (1/80 ratio). The exosomes contain abundant miRNAs of proliferation, angiogenesis, cell migration, immune response, and histone modification, which can affect the stomal cells. Moreover, the exosomes are incorporated by surrounding brain endothelial cells, which express respective mRNAs and induce the angiogenesis and proliferation of the recipient cells. A percentage of GBM-derived exosomes also harbor the EGFRvIII mutant [67]. miR-2276-5p presents low expression in the plasma exosomes of glioma patients compared to healthy controls, indicating that miR-2276-5p could serve as a diagnostic biomarker [95]. It seems that a high expression profile of the three miRNAs, miR-21, miR-124-3p, and miR-222, detected in exosomes is associated with the faster progression of high-grade gliomas following surgery [96]. MiR-301a levels in exosomes are reduced following surgical removal of primary glioma tumors and are increased after GBM relapse. MiR-301a activates FAK and AKT signaling by downregulating PTEN. MiR-301a exosome expression levels are independently associated with overall survival [97]. MiR-454-3p expression is decreased in glioma tissues, but its expression is upregulated in the exosomes of the same glioma patients. Both miRNAs were correlated with worst prognosis, while the restoration of miR-454-3p expression was shown to inhibit autophagy, cell migration, proliferation, and invasiveness [98]. The differential expression of exosomal miRNAs between cancer cells and normal cells has been also observed in other types of brain tumors, such as in pediatric high-grade glioma stem cells with a special focus on miR-1290 and miR-1246 [99]. There are also alternative RNA types, such as circular RNA detected in glioma-derived exosomes. circGLIS3 is upregulated in gliomas. Exosome-derived circGLIS3 induces Ezrin T567 phosphorylation and angiogenesis by endothelial cells [100]. In this context, the lncRNA ROR1-AS1 of glioma-derived exosomes induces tumor promotion in glioma cell lines through the suppression of miR-4686 [101].

Brain-derived exosomes are also enriched by several types of DNA cargo, such as micro-nuclear genomic DNA, mitochondrial DNA, oncogene amplifications, retrotransposons, and cytoplasmic and genomic DNA [102]. Aberrant DNA methylation is a known alteration which helps GBM classification. Genome-wide analyses in the EVs of GBM cells, matched culture cells, and tumors reflects the tumors’ methylation profile and classification [103].

In blood and in CSF, GBM exosomes can be detected via liquid biopsy (Table 2). Liquid biopsy, a new minimally invasive technique, can be performed at any time during the period of the disease and facilitates the monitoring of tumor progression [60,104]. The cargo of exosomes is related to the phenotype and genotype of their parent cells; thus, liquid biopsies might provide useful information related to prognosis, particularly in cancer [28,105,106].

A robust number of exosomes can be found in CSF, deriving from the tumor, whereas CSF is not contaminated with EVs from the blood, such as platelet-derived exosomes. On the other hand, blood samples are easier to collect, and not as invasive as the collection of CSF [69,104]. In addition, the study of Shao et al. showed that by using a nuclear magnetic resonance system in blood samples, it is feasible to detect GBM-shed microvesicles [107]. Exosomes contain molecules that can facilitate tumor progression and resistance to treatment by inducing a friendly microenvironment. In addition, exosomes have been highlighted as useful tools in the diagnosis of GBM as well as in prognosis by introducing more specific characterization of the tumor [60].

**Table 2:** Glioma-derived exosome biomarkers.

Study Type	Sample Type	Cargo (Biomarker)	Reference
Patients with glioma	CSF	miRNA-21	[108]
Patients with GBM	CSF	miR-21/miR-24/miR-103/miR-125	[109]
Patients with GBM who received TMZ treatment	CSF	miR-151a	[110]
In vitro (μNMR)	Blood	EGFR/EGFRvIII	[107]
GBM patients/In vitro	Tumor samples	EGFR/EGFRvIII	[111]
In vivo mice xenograft	Blood	PTRF	[112]
Patients with GBM (microfluidic chip)	Blood	MGMT, APNG	[113]
Patients with GBM	Serum	miR-574 3p/RNU6-1/miR-320	[114]
In vitro	Serum	miR-301a	[97]
Astrocytoma (grade II to IV) (TaqMan low-density array)	Serum	miR-15b-5p, miR-16-5p, miR-19a-3p, miR-19b-3p, miR-20a-5p, miR-106a-5p, miR-130-3p, miR-181b-5p, miR-208a-3p	[115]
Patients with GBM	Serum	miR-497, miR-125b	[116]
Patients with high-grade gliomas	Plasma	miR-221/222	[117]
Patients with glioma (grade I to IV)	Plasma	miR-21	[118]
In vivo glioma mice xenograft	Plasma	PDGFR, CAV1 IL-8	[85]
Chick embryo brain tumor	T98G cell line	L1CAM	[119]
In vitro (high-resolution MS)	U87 MG and LN229	ANXA1, ACTR3, APP, CALR, CTSD, ECM1, GAPDH, ITGB1, IGF2R, IPO5, MVP, PDCD6IP, PSMD2, PSAP	[70]
In vitro	U87 and U251 cell lines	miR-5096	[120]
In vitro	GBM U87 cells	miR-21, miR-23a miR-29a, miR-30a, miR-92b, miR-222, miR-221	[121]
In vitro	Glioma stem cells	miR-2	[83]
Patients with glioma	Tumor samples	*PTEN* mutations	[122]
Patients with GBM	Tumor samples	*IDH-1* mutant	[123]
Ex vivo	U87MG	Ndfifip1	[68]
In vitro/in vivo	U251	CXCR4, VEGF, MMPs (pro-MMP-9, pro-MMP-2, active MMP-2), plasminogen activators (tPA, uPA)	[124]

### 5.4. Current Approaches in Detecting Exosomal Biomarkers

Novel studies showed that surface-enhanced Raman spectroscopy (SERS) differentiates carcinogenic cells from normal cells [125]. The SERS method, an optical biosensor among nanomaterial-based techniques, has been proposed for label-free exosome detection and exosome detection with SERS-tags from various sources, including gliomas. It represents a powerful optical technique for biosensing and enabling further clinical diagnostics [125,126,127].

A custom-made platinum-black (Pt-black) SERS template has been created using a cost-effective electroplating fabrication technique. This template was designed specifically for the detection of aberrant (cancerous) exosomes. With the utilization of the Pt-black SERS template, an 83.3% sensitivity and a 95.8% specificity were achieved in distinguishing cancer-derived exosomes (obtained from 4T1 cells—a triple-negative breast cancer cell line) from exosomes derived from healthy fibroblast cells [128]. In the study by Agarwal et al., thirteen cancer cell lines (osteosarcoma, melanoma, breast tumor etc.) were sequenced via next-generation sequencing (NGS) and six miRNAs were identified, which were then studied and analyzed using Q-RT-PCR [129]. Cancer cells release circulatory miRNAs into the exosomal fraction, [130] and Cfa-miR-9 demonstrated consistent elevation in both NGS and PCR analyses, suggesting its potential as a promising diagnostic miRNA. The findings of this study propose the feasibility of identifying a pan-tumor specific miRNA through NGS and validating it via Q-RT-PCR, offering the potential for enhanced diagnostic efficiency [129].

The surface proteins present on exosomes derived from glioma cells can serve as reliable diagnostic biomarkers, providing valuable insights into the progression of gliomas [131]. Several advanced methods that are highly sensitive, employing real-time imaging, have been suggested to detect and measure these potential diagnostic membrane biomarkers. Among these, atomic force microscopy (AFM), localized surface plasmon resonance (LSPR), and surface-enhanced Raman scattering (SERS), represent some of the most advanced technologies that are already being employed for exosome detection [131,132]. AFM, a versatile scanning probe microscope, offers the ability of low-damage imaging for soft samples such as exosomes. Moreover, it has been utilized for the identification of biomarkers derived from glioma exosomes [133]. Recently, LSPR has also emerged as a valuable technique for detecting biomarkers exhibited by exosomes originating from diverse tumor types, including gliomas [134].

## 6. Exosomes Implication in Therapeutic Strategies Targeting Gliomas

Exosomes present structures surrounded by a lipid bilayer membrane with an aqueous core, which can accommodate both lipophilic and hydrophilic drugs [23]. Exosomes’ nano-range diameter and the fact that they can cross the BBB makes them promising candidates for gliomas’ therapeutic strategies (Table 3).

Exosomes can transfer drugs directly into the tumor, as well as decrease chemoresistance and reduce the systemic side effects of therapeutic agents [69]. When administered freely, Doxorubicin (DXR) and Paclitaxel (PTX) cannot cross the BBB, but upon packaging into vesicles, their delivery can be facilitated across the BBB, thus reducing tumor progression. Yang et al. used exosomes derived from U-87 MG glioma cells and brain endothelial cells (bEND.3) for the delivery of PTX or DXR across the BBB in a zebrafish brain tumor model [135]. Additionally, Sun et al. used exosomes to deliver curcumin and demonstrated its protective effects against septic shock induced by LPS in mice [136] while another study showed the positive effects of DEX electroporation in breast xenograft tumors after loading with doxorubicin [137].

A neutrophil-exosomes (NEs-Exos) delivering system for DXR as a treatment for glioma has been proposed. In vivo experiments on zebrafish and glioma mouse model showed that Nes-Exos can cross the BBB. Furthermore, these neutrophil-carrying exosomes not only cross the BBB, but they can also migrate chemotactically according to the inflammatory potential of tumor-infiltrating cells. The application of this DXR-delivering Nes-Exos system suppresses tumor development and extends the survival of glioma-bearing mice [138]. In the same context, another exosome-based delivering system has been constructed, which can simultaneously deliver nanoparticles for targeted imaging and diagnosis, as well as curcumin for targeted therapy. This combination has shown synergistic effects against glioma tumors in vivo [139]. The loading capacity and exosome features have also been documented for the delivery of PTX by exosomes. PTX incorporated by exosomes shows better efficacy against U-87 cells [140]. Exosomes generated from mesenchymal stem cells (MSCs) carry high levels of microRNA-29a-3p and suppress migration and vasculogenic mimicry, indicating the formation of VEGF-independent vasculature. A microRNA-29a-3p system can serve as anti-angiogenic supplementary treatment to resistant gliomas [141].

Natural exosomes are characterized by decreased yield; therefore, exosome mimics have been developed. These bioinspired nanovesicles have comparative drug-delivering capacity with natural exosomes; however, they have a 500-fold higher yield than natural exosomes. They show similar delivery capacity with a DXR cargo compared to natural exosomes [142].

It is known that human glioma-derived stem-like cells (GSCs) are involved in resistance mechanisms, including those associated with the alkylating chemotherapeutic agent, temozolomide. Treatment with temozolomide affects EVs generated from the GSCs of GBM patients. More specifically, EVs from GSCs become enriched with focal adhesions, cell adhesion, and extracellular matrix-receptor interaction molecules, thereby facilitating cell adhesion [143]. Furthermore, exosomes released by the reactive astrocytes of gliomas’ microenvironment are rich in O6-alkylguanine DNA methyltransferase (MGMT) mRNA, and this feature evokes transformation to a temozolomide-resistant phenotype [144]. High expression of miR-1238 also confers resistance to temozolomide [145]. On the other hand, miR-151a expression in temozolomide-resistant GBM cells sensitizes these cells to the drug through the inhibition of the X-ray repair cross-complementing 4 (XRCC4)-mediated DNA repair [110].

Exosomes derived from antigen-presenting dendritic cells can be further classified into subgroups based on their use in immunomodulation-based therapy, as delivery vehicles for anti-tumor nucleotides and as delivery vehicles for drugs. Dendritic cell-derived exosomes (DEX) have also been used for the treatment of brain tumors [146]. Immunotherapy based on dendritic cell vaccines is promising in preclinical models and in early clinical trials. Exosomes have been tested as a more efficacious antigen in co-delivery with α-galactosylceramide on a dendritic cells vaccine and show a strong induction of cytotoxic T lymphocytes, which are tumor-specific, thus suppressing immune tolerance [147].

Regarding the advancement of exosome therapy, it is important to select the appropriate manufacturing cells which produce exosomes, such as marrow stromal cells (MSCs) [148]. MSCs are isolated from posterior iliac crests and further processed through the aspiration of bone marrow. This is promising since exosomes can be produced from the patient’s own cells. Moreover, DEX therapy has shown promise for their development and in clinical testing for non-small cell lung cancer and metastatic melanoma treatment [149,150,151]. It has also been reported that DEX-loaded dendritic cells from glioma cells derived chaperone-rich cell lysate-loaded dendritic cells, promote T-cell activation, and exhibit anti-tumor effects in mice with intracranial glioma [152].

**Table 3:** Potential exosomes therapy/exosomes drug delivery.

Type of Study/Model	Exosomal Cargo	Effect In Tumor	Reference
In vivo/xenograft tumor in nude mice	miR-375	Increases apoptosis, suppresses SLC31A1 proliferation/migration/invasion in glioma	[153]
In vivo U251 cells	miR-199a	Down-regulates AGAP2/inhibits glioma progression	[154]
In vitro/vivo GL261 glioma cells	CRCL	Modulates Cbl-b/c-Cbl signaling/anti-tumor activity	[152]
Patients with glioma	miR-454-3p	Suppresses cell proliferation, invasion, migration, and autophagy in gliomas	[98]
In vitro/vivo glioma U87	miR-133b	Inhibits EZH2 and Wnt/β-catenin and represses proliferation/invasion/migration in glioma	[155]
In vitro/vivo U-87 MG xenograft nude mouse	miR-584	Inhibits glioma growth	[156]
In vitro/ex vivo rodent model xenograft 9 L glioma	miR-146b	Inhibits glioma growth	[157]
In vitro (U87 and X12 GBM) In vivo xenograft nude mouse	miR-1	Inhibits glioma growth	[158]
In vitro (GSC26-28 GSC6-27) In vivo glioma xenograft mouse	miR-124	Dysregulates cell metabolism	[81]
In vivo embryos zebrafish model	Paclitaxel (PTX)/Doxorubicin (DXR)	Delivery anticancer drugs	[135]
In vitro U87MG In vivo glioma mice xenograft	KLA peptide LDL/Methotrexate (MTX)	Delivery of anticancer drugs and targeted peptides for therapy	[159]

## 7. Conclusions

It has been established that exosomes play a notable role in gliomas pathogenesis. They can be produced and secreted by cancer cells and therefore facilitate interactions between cancer cells and the tumor microenvironment. In this way, exosomes facilitate cell invasion and migration, cell proliferation, cancer cell stemness, angiogenesis, immune tolerance, malignant transformation, and ultimately, resistance to treatment.

Exosome-targeted therapy is presently under ongoing development that is continuously evolving. Engineered exosomes present promising tools that can enhance or improve the conventional drug-delivery systems (Figure 1). Their ability to cross BBB is attributed to their small size, their flexibility, and the presence of adhesive proteins on their surface. Moreover, the endogenous production along with their encapsulation in lipid bilayer minimizes their toxicity and immunogenicity while increasing their stability in peripheral blood [160,161]. Exosomes can be used as potential therapeutic and diagnostic tools because they can mediate intercellular communication during brain tumor development [162,163,164], especially in the context of the challenging treatment of gliomas [165]. Exosomes may serve as delivery systems for various therapeutic agents. They also represent the biomarkers of therapeutic responses, through which resistance to treatment can be monitored with minimally invasive methods (Figure 1) [166]. For clinical applications, the intravenous administration or intranasal administration of exosomes may be achieved to enable the targeting of tumor cells. Exosomes transport may be accelerated by tumor-targeting membrane peptides and might be viable when delivered intravenously or intranasally to brain tumors [167,168]. Future studies should focus on advanced methods that will ensure the reliable detection of exosomes and exosomes cargo, as well as the potential of using exosomes as targeting delivery systems for therapy in gliomas.

## Figures and Tables

**Table 1:** Exosome isolation methods.

Method/Technique	Advantage	Disadvantage	References
Ultracentrifugation (UC)	Easy application	Low discrimination (demanding in low density and small sizes)	[50,51,52,53,54]
Ultrafiltration (UF)	Fast/low cost	Low efficacy/specificity	[55]
Immunoaffinity isolation	High purity	High cost/specific antibody	[51,56]
Polyethylene glycol (PEG) precipitation	Simplicity/relatively low cost	Variable efficiency/ inability to separate similar-sized particles	[51]

## Data Availability

Not applicable.
